# Editorial: Acute and chronic effects of sub-concussion on brain function

**DOI:** 10.3389/fneur.2022.1121589

**Published:** 2023-01-06

**Authors:** Andrew P. Lavender, Ryusuke Takechi, Keisuke Kawata, Kerry Peek, Thomas George Di Virgilio

**Affiliations:** ^1^Institute of Health and Wellbeing, Federation University Australia, Ballarat, VIC, Australia; ^2^Curtin Health Innovation Research Institute, Curtin University, Perth, WA, Australia; ^3^School of Public Health, Indiana University, Bloomington, IL, United States; ^4^Discipline of Physiotherapy, Faculty of Medicine and Health, University of Sydney, Sydney, NSW, Australia; ^5^Faculty of Health Sciences and Sport, University of Stirling, Stirling, United Kingdom

**Keywords:** sub-concussion, concussion, mild traumatic brain injury (mTBI), blood-brain barrier, Mechanically Induced Neurophysiological Disruption

In recent decades, repeated minor head impacts resulting in mild traumatic brain injury (mTBI) or sub-concussion due to its cumulative nature, has attracted attention from clinicians and scientists. Sub-concussive head impacts are common and can arise from participation in popular sports, such as soccer, Australian football, American football, and rugby, from accidents (e.g., car crash and fall), and from injuries during military service. Although acute clinical signs are not present, chronic symptoms such as neurocognitive deficits and psychomotor dysfunction years and decades following repeated sub-concussive head impacts have been reported. This indicates the serious consequences of “silent” mTBI.

This *Frontiers in Neurology* Research Topic brings together novel research on sub-concussion and its acute and chronic effects through animal models and clinical studies. Seven articles provide an insight into the pathophysiological underpinning, assessment, and treatment of such injuries.

An important consequence of mTBI is change to brain anatomy and physiology at the cellular level. Structural damage to the blood brain barrier (BBB), due to a single head impact, can occur. Optimizing tools and developing new technologies for investigating changes in the brain resulting from such head impacts are crucial for advancing our understanding of functional changes in brain activity induced by mTBI (Leaston et al.). This study examined the feasibility of “qualitative ultrashort time-to-echo contrast enhanced” or QUTE-CE for monitoring early pathology and vulnerability of the BBB following a single or repeated mTBI. Structural damage and BBB permeability in rats were assessed at baseline and within an hour after the head impact using anatomical images and QUTE-CE biomarkers. A change in BBB permeability was reported following the second and third impacts, affecting multiple brain regions supporting the concerns among healthcare professionals of repeated mTBI.

Acute stage assessment of mTBI injury severity and sub-clinical head impacts are vital as these may result in persistent neuropsychiatric effects, especially, when impacts are repeated. This suggests a need for objective assessments to support acute clinical diagnosis and inform predictions of long-term outcomes. Neurocognitive testing combined with biomarker assessment can be used to diagnose brain injury when imaging is not sufficient. A study assessed the use of the “HEAD injury Serum markers and Multi-modalities for Assessing Response to Trauma” (Head SMART II) to develop a blood biomarker for evaluating acute traumatic encephalopathy (Peacock et al.). The BRAINBox TBI Test incorporates proteomics with clinical assessments to identify and define pathologic evidence of neurocognitive impairments. This has important clinical applications in diagnosing brain injury and providing an objective assessment of the patient's risk of developing post-concussive symptoms.

Changes in behavior and cognition have been observed following repeated sub-concussion (Boucher et al.). Repeated mild traumatic injury (rmTBI) impacts were designed to replicate an athletes' experience, such as soccer heading or tackling in rugby through matches or practice sessions, in a mouse model. Behavioral measures, serum biomarkers, cortical protein quantification, and immunohistochemistry were assessed following rmTBI at various frequencies. Serum biomarker analysis showed that GFAP and NF-L did not change, and decreases in IL-1β and IL-6 were observed in serum and cortical tissue, indicating a downregulation of inflammatory pathways. These findings provided insights in potential mechanisms of repeated sub-concussion.

An important research endeavor is to find ways to ameliorate injuries in athletes to improve safety and recovery in sport. The article by Raikes et al. assessed the neuroprotective effects of docosahexaenoic acid (DHA) and eicosapentaenoic acid (EPA) supplementation against repetitive sub-concussive head impacts (RSHI). American football players were randomized into a treatment group who received DHA and EPA or a placebo group receiving oleic acid and linoleic acid. Neuroimaging data were obtained at baseline and post-season. Deterministic tractography using quantitative anisotropy showed an increase in structural connectivity in ascending cirticostriatal fibers and a decrease in long association and commissural fibers in the DHA+EPA group compared with the placebo group, indicating protective effects against axonal damage.

A greater awareness of sport-related concussion has led researchers to investigate ways to optimize diagnosis and monitoring for safe return-to-sport (RTS). The study by Vorn et al. investigates the plasma proteomic profiling related to RTS following a sport-related concussion. This study collected blood samples from 140 athletes within 48 h of injury, and a highly multiplexed proteomic technique was applied to target 1,305 proteins in plasma samples. Thirty two proteins were upregulated while 55 were downregulated in those who took more than 14 days to recover compared with those who recovered within 14 days, suggesting that large-scale plasma proteomic profiling across acute to subacute time periods may predict long-term outcome.

While animal studies are important, translating the knowledge into human research is a vital next step. The study by Smirl et al. assessed heart rate variability (HRV) and cardiac baroreceptor sensitivity (BRS) metrics in elite soccer players following 40 headers, compared with body contact control, and non-contact sham conditions. ECG and finger photoplethysmography characterized acute HRV/cardiac BRS, before and after each condition. The SCAT3 was also applied. While heart rate increased from pre to post heading, HRV metrics in the time domain and frequency domain as well as cardiac BRS remained unchanged. Interestingly, SCAT3 score increased compared with the control and sham conditions. The study showed that, while SCAT3 scores were affected by a bout of controlled heading, no changes were detected in autonomic function.

“Sub-concussion” has been described as “a cranial impact that does not result in a known or diagnosed concussion on clinical grounds” (Lavender et al.). This definition addresses the clinical outcomes of the incident. It has also been defined as “repetitive subconcussive impacts (RSHI)” which adds the aspect of repetition as an important component. A lack of precision in describing sub-concussion has clinical and diagnostic implications. The article by Lavender et al. attempts to clarify the discussion by introducing the term “Mechanically Induced Neurophysiological Disruption (MIND)” to make the distinction between the incident of an impact and the resultant physiological responses. MIND is described as a sudden or ballistic low magnitude movement of the brain that disrupts neurophysiological function and connectivity resulting in asymptomatic changes with potential to affect neural pathway function if performed repeatedly for long periods. This definition encompasses the repeated nature of the events that lead to anatomical and physiological modulation of brain function.

Collectively, the articles in this Research Topic make a significant contribution toward better understanding of the effects of repeated sub-concussive head impacts. This Research Topic can impact grass roots through to elite sporting levels and military exercises, making sport, and military training safer.

## Author contributions

All authors listed have made a substantial, direct, and intellectual contribution to the work and approved it for publication.

